# Self-rated health among Mayan women participating in a randomised intervention trial reducing indoor air pollution in Guatemala

**DOI:** 10.1186/1472-698X-8-7

**Published:** 2008-06-05

**Authors:** Esperanza Díaz, Nigel Bruce, Dan Pope, Anaité Díaz, Kirk R Smith, Tone Smith-Sivertsen

**Affiliations:** 1 Department for Public Health and Primary Health Care, University of Bergen, Norway; 2 Division of Public Health, University of Liverpool, UK; 3 Center for Health Studies, Del Valle de Guatemala University, Guatemala; 4 Division of Environmental Health Sciences, University of California, Berkeley, USA

## Abstract

**Background:**

Indoor air pollution (IAP) from solid fuels is a serious health problem in low-income countries that can be alleviated using improved stoves. Although women are the principal users, few studies have investigated the self-assessed impact of the stoves on their health and lives.

**Methods:**

This study was conducted in rural highland Guatemala, involving 89 intervention and 80 control Mayan Indian young women (mean 27.8 years, SD 7.2). Outcomes were assessed after approximately 18 months use of the new stove. Our objectives were to compare self-rated health and change in health among women participating in a randomised control trial comparing a chimney stove with an open fire, to describe impacts on women's daily lives and their perceptions of how reduced kitchen smoke affects their own and their children's health.

**Results:**

On intention-to-treat analysis, 52.8% of intervention women reported improvement in health, compared to 23.8% of control women (p < 0.001). Among 84 intervention women who reported reduced kitchen smoke as an important change, 88% linked this to improvement in their own health, particularly for non-respiratory symptoms (for example eye discomfort, headache); 57% linked reduced smoke to improvement in their children's health, particularly sore eyes.

**Conclusion:**

Women's perception of their health was improved, but although smoke reduction was valued, this was linked mainly with alleviation of non-respiratory symptoms like eye discomfort and headache. More focus on such symptoms may help in promoting demand for improved stoves and cleaner fuels, but education about more severe consequences of IAP exposure is also required.

## Background

Indoor air pollution (IAP) from biomass fuels used for cooking and heating is a global health problem impacting particularly on poor people in rural areas of low-income countries. Around 1.5 million premature deaths are attributed to biomass-fuel IAP each year [1]. Solutions to reduce IAP levels, and therefore improve health, include use of cleaner fuels, improved stoves, and better ventilation practices [2]. Studies have shown that improved stoves with chimneys can reduce IAP levels by 40–60% over extended periods [3-5]. Cost-effectiveness [6] and cost-benefit [7] analysis support the promotion of improved stoves to reduce exposures within biomass fuel-using households, until universal access to cleaner fuels becomes achievable.

Despite the benefits of improved stoves and some major program successes [8], many developing-country households fail to adopt improved stoves. Attempts have been made to learn from past experiences [9,10]. Reasons are complex, and often locally specific, but the lack of involvement of women in the project cycle has been identified as one major factor contributing to poor uptake and sustainability [11]. Yet, there are few published studies [12,13] to date that incorporate women's perspectives on the introduction of improved stoves in populations previously using open fires.

RESPIRE (Randomised Exposure Study of Pollution Indoors and Respiratory Effects) Guatemala was the first randomised controlled trial designed to study the health effects of reducing IAP, achieved using locally produced chimney stoves (*Planchas*). The main outcomes studied were acute lower respiratory infections (ALRI) in children under 18 months [14], and respiratory health of their mothers [15]. The *Plancha *significantly reduced personal exposure to IAP by nearly 45% [16]. A secondary outcome included assessing the self-perceived impact of the *Plancha *on the lives and health of the women. Self-rated health is an important component of quality of life [17], and a central measure of health status that predicts declines in functional ability and survival [18,19], and also affects the demand for health services [20].

The aims of this paper are (i) to compare self-rated health and change in health between 89 intervention and 80 control women taking part in RESPIRE, (ii) to describe the impact of the stoves on daily life, and (iii) to explore women's perception of a link between IAP and their own health and that of their young children.

## Methods

RESPIRE was carried out in the Guatemalan highlands among indigenous Mayan women. The local language is Mam, and wood is the primary cooking and heating fuel. Women usually carry their youngest child at their back while cooking for the family. The study ran from October 2002 to December 2004 and has been described in detail elsewhere [15,21].

**Figure 1:**
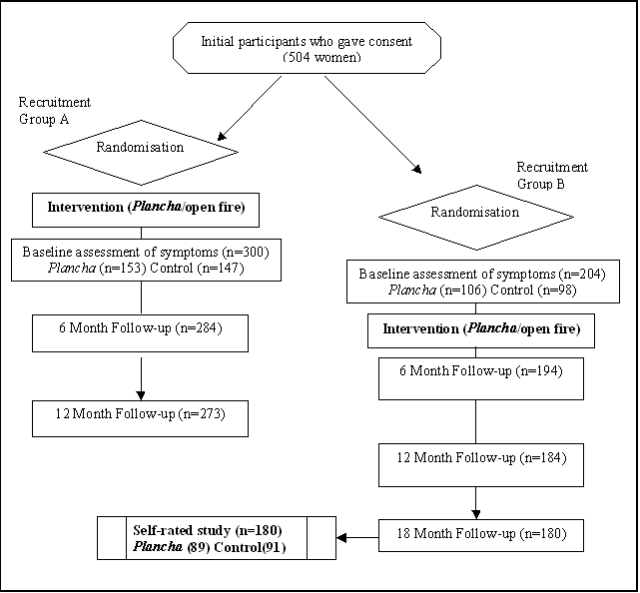
Flowchart of RESPIRE study, and self-rated study.

After a local census and oral consent, 504 women using open fires for cooking and heating, who were either pregnant or mothers to a child aged 4 months or younger, were randomised to receive, free of charge, an improved stove (*Plancha*) or to continue open fire use. The *Plancha *has a metal flue that expels most smoke out of the house. This type of stove has been shown to deliver substantial reductions in kitchen IAP levels [22] and in personal exposures – both in this study [16] and adjacent communities [23]. All control women were offered a free *Plancha *at the end of the study. To avoid extending the geographical study area, participants were recruited over two periods: the first between October and November 2002 and the second between April and May 2003 (Fig. 1). Intervention women were carefully instructed in the use and maintenance of the stoves but did not receive any other health advice that differed from controls.

Baseline information, including demographic, socio-economic and health questions, was obtained from all households using interview-led questionnaires during home visits (Baseline questionnaire -BQ-). Women also took part in several assessments of symptoms, measurements of exposure, and lung function test (six-monthly health assessments -HA-). The last of these health assessments occurred between September and November 2004. Results describing the women's respiratory health at baseline and the impact of the stove on non-respiratory symptoms have been published [15,16].

Also, between September and November 2004, a final interview was carried out in the second recruitment group (180 women). The interview was developed in Spanish, translated into Mam and backtranslated, and was designed to collect the following information:

A. Self-rated health was elicited by the question: *"Generally speaking, how is your health: good, average or poor?" *For logistic regression analyses, the answers were combined into two categories: (1) good, and (2) average and poor. Change in health during the study was elicited by the question: *"How is your health now compared to the beginning of the study: better, the same or worse?"*

B. Intervention women were asked: *"In which way, for better or worse, has the Plancha changed your life, if there has been any change at all?" *Based on previous experience during the study and on pilot work, a list of possible answers was developed, with space to record other responses. Fieldworkers only ticked a particular item if the woman mentioned it without prompting.

C. Intervention women who mentioned "reduction of smoke in the kitchen" in B, were also asked: (i) *"Do you think that the smoke reduction has influenced your own health in any way?" *and (ii) *"Do you think that the smoke reduction has influenced your children's health in any way?" *If a woman answered yes to either question, she was asked to explain in which ways the smoke reduction had influenced her own or her children's health, and the fieldworker wrote down the symptoms reported using free text and with multiple responses allowed.

This questionnaire was piloted in 37 women from the first recruitment group. The interview was conducted in Mam, and administered by two bilingual (Spanish and Mam) fieldworkers, randomly assigned to intervention and control women.

At the 18 month assessment, the second recruitment group comprised 91 control and 89 intervention women. Of these, eleven control women received a *Plancha *before the self-rated health assessment, according to the original protocol (seven either had a miscarriage or their children had died, and four had left the study). These women had used the *Plancha *for a shorter period than intervention women (mean of 4.96 (SD 2.47) months vs. mean of 16.31 (SD 0.60) months respectively). In order to perform an intention to treat analysis (ITT), results from all 91 control women were included in analysis of the first part of the questionnaire, whilst only answers from the 89 intervention women were included in analyses of the rest of the questionnaire.

All analyses were performed using SPSS v13 [24]. Chi-squared tests were used for the ITT analysis of the first two closed questions to test for differences between intervention and control groups. Information on respiratory and non-respiratory symptoms from the last HA was merged with data from the self-rated health interview. A binary logistic regression model was used to study the independent effect of reporting symptoms at the 18 month HA on self-rated health, with adjustment for age (in years), pregnancy, intervention status and fieldworker. For these analyses, three ordinal variables were created: "Any symptom" based on the total of all symptoms reported by each woman in the HA interview (from 0 "no symptoms" to 7 "all the symptoms including cough, phlegm, wheeze, tightness in the chest, headache, back pain (due to cooking position) and eye discomfort"); in the same way, "Respiratory symptoms" (scored from 0 to 4) and "Non-respiratory symptoms" (scored from 0 to 3) were created.

The adult component of RESPIRE was approved by the Research Ethics Committees at the Universities of Bergen, Liverpool and Del Valle Guatemala.

## Results

Baseline (BQ) characteristics of the study participants are shown in Table 1, by randomisation group. Women were young (mean age 27.8 years, SD 7.2, range 15–44 years), and none smoked. A high percentage (68.9%) of the women were pregnant at baseline, but only 12.8 percent (12.6% in the intervention group and 13.6% in the control group) were pregnant at the time of final (18 month) interview. At baseline, more than two thirds of the study women reported one or more acute symptoms (eye discomfort, headache, cough, back pain) while cooking. Women reported a mean of 2.9 hours per week (SD 2,14) gathering wood, and spent a mean of 120 quetzals (US $16.3) (SD 68) per month on this fuel. The main lighting source for both the intervention and the control group was electricity. There were no significant differences between intervention and control households at baseline, indicating balanced randomisation.

**Table 1:** Baseline characteristics for intervention (*Plancha*) and control (open fire) groups.

	*PLANCHA*	*OPEN FIRE*
Number of women	89	91
Characteristics of the women:		
Mean age in years (SD)	27.6 (7.7)	28.3 (6.7)
Not attended to school (%)	32 (36.0)	36 (39.6)
Pregnant at baseline	62 (69.7)	62 (68.1)
Mean number of children < 12 years (SD)	3.7 (1.9)	3.8 (1.5)
Smokes (%)	0 (0.0)	0 (0.0)
Cough while cooking (%)^a^	59 (66.3)	61 (67.0)
Headache while cooking (%)^b^	64 (71.9)	71 (78.0)
Sore eyes while cooking (%)^c^	78 (87.6)	78 (85.7)
Back pain while cooking (%) ^d^	54 (60.7)	60 (65.9)
Household characteristics:		
Wood as main fuel (%)	89 (100)	91 (100)
Cooking area in a separate structure (%)	67 (75.3)	68 (85.0)
Relative smokes inside (%)	17 (19.1)	22 (24.2)
Mean number cigarettes husband (SD)	1.2 (0.4)	1.1 (0.2)
Kitchen separate from bedroom (%)	70 (78.7)	79 (86.8)
Asset index^ε^:		
0	8 (9.0)	15 (16.5)
1	47 (52.8)	50 (54.9)
2	23 (25.8)	18 (19.8)
3	9 (10.1)	8 (8.8)
> = 4	2 (2.2)	0 (0.0)

a When you are cooking, does the smoke make you cough or irritate your throat? (yes = sometimes/always)

b When you are cooking or immediately after, do you get a headache? (yes = sometimes/always)

c When you are cooking, do your eyes get irritated? (yes = sometimes/always)

d When you are cooking, do you get back pain? (yes = sometimes/always)

e Sum of goods (radio, TV, refrigerator, bicycle, motorcycle and car) a family has. From 0 for not having any of them, to 6 for having all of them

At 18 months, a total of 76 (85.4%) intervention women and 71 (78.0%) control women rated their health as good (p 0.141) (Table 2). After a mean of 16.31 months (SD 0.60) using a *Plancha*, 52.8% of intervention women reported that their health had improved, compared to 24.2% of control women (p < 0.0001). No woman reported a decline in health since beginning the study.

**Table 2:** Self-rated health and self-assessed change in health for women using a *Plancha *compared to open fire.

	*Intervention (89) no.(%)*	*Control (80 no (%))*	*p*
Generally health is^a^			
Good	76 (85.4)	61 (76.3)	0.141
Average	10 (11.2)	10 (12.5)	
Poor	3 (3.4)	9 (11.3)	
Health after the study is^a^			
Better	47 (52.8)	19 (23.8)	< 0.0001
Equal	42 (47.2)	61 (76.3)	
Worse	0	0	

a Text of questions: "Generally speaking, how is your health?" and "How is your health now compared to the beginning of the study?"

Logistic regression revealed that the odds of rating health average or poor, compared with good, significantly increased with increasing number of symptoms (Table 3), especially for non-respiratory symptoms. The odds of rating health average or poor also appeared to increase with age in years, although not significantly (p = 0.07).

**Table 3:** Association between symptoms at 18-month assessment and self-rated health.^a^

	OR^b^	95% CI^c^
Any symptoms at 18 months	1.35	1.02–1.78
Fieldworker	1.00	0.96–1.03
Intervention status	0.74	0.32–1.71
Age (years)	1.05	0.99–1.11
Pregnancy	1.39	0.45–4.23
		
	OR^a^	95% CI^b^
Respiratory symptoms at 18 months	1.33	0.84–2.08
Fieldworker	1.00	0.97–1.04
Intervention status	0.63	0.28–1.43
Age (years)	1.05	0.99–1.12
Pregnancy	1.41	0.47–4.28
		
	OR^a^	95% CI^b^
Other symptoms at 18 months	1.60	1.03–2.49
Fieldworker	1.00	0.96–1.03
Intervention status	0.77	0.33–1.81
Age (years)	1.05	0.99–1.12
Pregnancy	1.37	0.45–4.13

a Same analysis of the whole sample including different variables in the model (either any symptom or respiratory symptom or other symptoms).

b Odds ratio (OR)

c 95% confidence intervals (CI)

When women were asked if and how the *Plancha *had changed their lives, nearly all reported, unprompted, that the smoke had been reduced in the kitchen, which was described as more comfortable, clean and with less of a smell of smoke, resulting in a place where women were proud of working (Table 4). Most women talked about improvement in cooking tasks, including better posture (from squatting to standing) with the *Plancha*, which could also be used as a working surface, and permitted shorter cooking time (allowing cooking with more than one pot at a time). The *Plancha *was also reported to reduce worry about children getting burned, and to save wood and money. One third of women reported that having a *Plancha *improved the family social status.

**Table 4:** Reported advantages and disadvantages of using a *Plancha*.

*All women using Plancha n = 89*			
**Advantages**	**n**	**Disadvantages**	**n**

Less smoke	84	Colder in the kitchen	7
Easier everyday work		Difficult to get light from *Plancha*	2
Better position	73	Cannot use light from fire	3
*Plancha *can be used as surface to work	13	More time cutting wood into pieces	13
Shorter cook time	59	Longer time cooking	6
Cleaner clothes	37	Difficult cooking with big clay pots	11
Less cloth to wash	3	Difficult cooking food for animals	9
Cleaner pots	14		
Cleaner children	24		
Cleaner skin	16		
Comfort			
Warmer inside the house	4		
More comfortable kitchen	28		
Less smoke smell in the house	3		
Less smoke smell in the clothes	4		
Less smoke smell in the hair	3		
Potential for saving resources			
Save wood	32		
Save money	3		
Social benefits			
Improved social status	29		
Proud to work in the kitchen	17		
Kitchen is now a meeting point	1		
Health benefits			
Less worried about burns in children	48		

Reported negative aspects relating to the *Plancha *included a longer required time for cutting wood into smaller pieces than typically used in open fires, difficulties cooking with big clay pots and with cooking animal food, lower kitchen temperatures, longer cooking time and reduced light from the fire (Table 4).

Among the 89 intervention women, 84 mentioned a reduction of smoke resulting from the stoves. Of these, 74 reported that this reduction had influenced their own health, and 48 that reduction had influenced their children's health in some way. Self-reported improvements in health included a reduction of eye discomfort (52 women), headache (15 women) and throat discomfort (9 women). Most (45) of the 48 women who stated that the smoke reduction had an effect on their children's health, explained this in terms of reduced eye discomfort. By contrast, very few women mentioned a reduction in respiratory symptoms as the explanation for improved health, either for themselves (4 women) or for their children (1 woman).

## Discussion

Despite the poor and difficult conditions in which they live, most women in the study rated their health as good. More women in the intervention group compared to control women reported their health as good after 18 months (p = 0.141), and significantly more (p < 0.001) reported that their health had improved during the trial. Nearly all intervention women reported a reduction of kitchen smoke when asked whether and how the *Plancha *had changed their lives. Most thought that the smoke reduction had improved their own health, but only half thought that it had improved their children's health. Reductions in non-respiratory symptoms (sore eyes, headache, etc) were reported more often than respiratory symptoms, in terms of the explanation for improved health resulting from reduced smoke levels.

To our knowledge, this is the first study on IAP in a developing country that specifically includes self-rated health and change in health as outcomes for assessing the impact of an intervention on the lives of the participants. Women in developing countries, especially in the rural communities, have a long tradition of using open fires, not easy to change. It is therefore important to identify and understand the changes that women report in their everyday lives associated with using the stoves, as this is likely to be an important factor for their use, maintenance and continued promotion within communities.

To obtain the information we sought, different approaches could have been used. The possibility of using a validated measurement instrument, for example the SF-36 [25], was considered. Unfortunately, few questions were appropriate for our study population, either because the women were not familiar with the activities or the concepts used or because the grading of answers into five responses, that proved too complicated for translation into Mam. Instead, we developed an *ad hoc *questionnaire with the two first questions similar to those used in other self-rated health studies [26-31]. The questions required further adaptation and translation into Mam and had only three possible answers, compared to four [20] or five [17] generally used in the study of self-rated health and change in health [29]. The answers to self-rated health questionnaires, however, are often re-categorised in two or three categories for analysis, becoming more similar to ours.

Few studies have examined individual transitions in self-rated health. Hass et al reported smaller changes in self-reported health status during the course of pregnancy than they did in physical function and vitality [32]. Leinonen et al [29] did not find any change in self-rated health when the same question was asked at two different time points, five years apart, in a elderly population without any specific intervention. When the same individuals were asked about *change *in their health status in the past five years, however, nearly half said that health had become worse. Also in our study, despite the fact that most of the women rated their health as good at the end of the trial, significantly more women in the intervention group considered that their health had improved during the study compared to the control women. Since women were randomised to either intervention or control groups, this is most likely an effect of the *Plancha*.

One potential for uncertainty in interpretation of these results is the possibility that there might be a greater tendency to give socially approved responses among one of the groups. However, there are reasons to think that our findings represent real changes in the women's perceptions. Firstly, associations observed between symptoms from the 18-month HA and the level of self reported health were found to be strongest for non-respiratory symptoms, consistent with women's unprompted explanations of health improvements resulting from smoke reduction. Since the 18-month HA and the self perceived study were independent to a degree (they were conducted some weeks apart for each household and two completely different questionnaires were used), this represents internal consistency. Secondly, at 18 months the intervention homes already had their *Planchas*, while control homes were about to receive theirs. Thus, controls would arguably be as motivated as intervention group women to please the investigation team in their answers about health status. Thirdly, there were two different fieldworkers that interviewed the women. Although these fieldworkers were local women who shared the ethnic background with the participants, the possibility of a bias in the interpretation of the participants' responses exists. However, the fieldworkers were randomly assigned to the intervention and control group, which reduces the likelihood of this bias.

The percentage of women that were pregnant decreased substantially during the study period, approximately 70 percent to13 percent. Although it is known that women experience substantial changes in health status during and after pregnancy, it seems that self-rated health status exhibits smaller changes over the course of pregnancy than other types of health assessment [32]. Also, as the percentages of pregnant women in the control and intervention groups were balanced, this issue is not likely to alter our results, although it should be taken in consideration when comparing our results with other populations.

Most of the women in this study reported having good health, although many reported suffering daily from symptoms. The answers to self-rated health questions, however, are based on knowledge that is built up over the life course from somatic experience, health care encounters, information about symptoms and diseases, and from social networks [17,30,33]. One consistent difference frequently noted in earlier studies (mostly on elderly people) was that, where self-rating and physicians' ratings were discrepant, respondents tended to rate their health as better than the doctors did [19]. Indeed, it has been hypothesized [34] that the more prevalent an objectively abnormal physical or emotional condition is in one's reference group, the less likely one is to attribute great significance to it. If this is true, the high prevalence of symptoms like eye discomfort, headache or cough reported in the study population, that was representative of women's reference groups, provided norms against which each woman evaluated her own health.

The question of how comparable self-rated health ranks are between ethnic groups has been raised [28]. A study about the comparability of self-rated health across cultures between Finland and Italy [31], found that the correlation between self-rated health and symptoms was similar for both cultures, although the cut-off point for rating their health as good differed between the two cultures. The significant association that we found between self-rated health and symptoms corroborates these results in a different population and strongly supports the appropriateness of this question. Still, as the reference point to define self-rated health as "good" may be different in different cultural environments, comparisons of the level of self-rated health across cultures should be made with caution.

Some of the impacts of the *Plancha *have been described earlier by Shaller [13] among women using the lorena stove in Guatemala and by Bates et al [10] among women in Kenya using different interventions to alleviate IAP. Other benefits more specifically related to the *Plancha *use were identified in our study, such as the possibility of saving time by carrying out two cooking tasks simultaneously, also reported by Khushk et al [12], the possibility to use the stove as a working surface, and the saving of wood. Also, in accordance with Shaller [13], we identified some disadvantages linked to the stove use, which need to be addressed appropriately to ensure women continue using their new stoves.

Most of the women in the intervention houses reported, without prompting, reductions in kitchen smoke, and awareness of the relationship between this reduction and their own health. Indeed, the symptoms that they talked about (eye discomfort and headache) were the same as those described by women in Kenya [10]. These symptoms were also found to be most strongly associated with self-rated health and were significantly reduced among women using *Planchas *in the RESPIRE study [16]. Although respiratory symptoms related to IAP are a more important cause of morbidity and mortality among these women, it is the non-respiratory acute symptoms that are perceived as a greater burden in their daily lives. Thus, the reduction of these symptoms should be given due attention when promoting the dissemination of improved stoves.

More women considered the *Plancha *to be beneficial to their own health compared to their children's health. This difference might have several explanations. Indigenous women in Guatemala have used open fires for many centuries, and do not usually appear to think of the smoke as related to serious illness [35]. In this context, the relationship between the smoke and children's illness might be difficult to detect [36]. In fact, the only symptoms the women talked about regarding children were those that were easily recognisable to an observer: tears and burns. It has been hypothesised [37], however, that a household needs to have a certain level of knowledge about the relationships between fuel use and health to demand an intervention designed to reduce IAP. Thus, our results draw attention to the importance of educational programs run parallel to dissemination programs, to broaden the areas of perception of health benefits of improved stoves.

## Competing interests

The authors declare that they have no competing interests.

## Authors' contributions

ED drafted and wrote the manuscript and performed the statistical analysis. TS–S and NB conceived the study, and participated in its design and coordination and helped to draft the manuscript. DP helped to draft the manuscript. AD organized data collection and participated in study design. KRS conceived the principal study and helped to draft the manuscript. All authors read and approved the final manuscript.

## Pre-publication history

The pre-publication history for this paper can be accessed here:


